# Editorial: Compassion and compassionate leadership in the workplace

**DOI:** 10.3389/fpsyg.2022.1074068

**Published:** 2022-12-09

**Authors:** Paula Benevene, Ilaria Buonomo, Michael West

**Affiliations:** ^1^Department of Human Sciences, Libera Università Maria SS. Assunta, Rome, Italy; ^2^Department of Organization Work and Technology, Lancaster University, Lancaster, United Kingdom

**Keywords:** compassion, compassionate leader, engagement (involvement), wellbeing, organizational wellbeing

How can we make workplaces radically better for people across the world? A fundamental human orientation is compassion that underpins belonging and psychological safety. Developing and sustaining compassionate cultures is essential for transforming workplaces to promote human happiness and wellbeing.

Compassion in an organizational context can be understood as having four components: (i) attending, (ii) understanding, (iii) empathizing, and (iv) helping (Atkins and Parker, 2012). In the context of an interaction between people, compassion involves:

1. Attending—paying attention to others and noticing their suffering;
2. Understanding—understanding what is causing others' distress by making an appraisal of the cause, ideally through a dialogue;
3. Empathizing—having a felt empathic response, to some extent mirroring others' distress;
4. Helping or serving—taking thoughtful, skilled, and appropriate action to help relieve others' suffering or help them cope more effectively.

The main objective of building compassionate cultures at work is creating conditions where all are supported to have the best and most fulfilling work lives possible. Compassion at work includes being effective in pursuing a commitment to embodying our values in our work, which requires shared direction, alignment, and commitment. Compassion also implies inclusion, which means working together to include all, regardless of professional background, opinion, skin color, sexuality, religion, or gender. Compassion implies sharing power by encouraging collective leadership, where all feel they have some leadership influence. Finally, compassion requires us to work together to develop a climate of shared purpose, prioritizing high-quality outcomes (for the organization, those we serve, and those who work in the organization) in our workplaces rather than just in our individual areas of responsibility.

Compassionate leaders have a crucial role in promoting and delivering compassionate cultures at work. They embody both a sensitivity to the challenges that those they lead face and a commitment to help them respond effectively to those challenges and thrive in their work process.

A compassionate leader typically shows four key behaviors, mirroring the dimensions of compassion at work described above:

- Attending: being present with and attending to those they lead (West et al., 2015; West, 2021);
- Understanding: understanding through dialogue with those they lead (West et al., 2015; West, 2021);
- Empathizing: being able to feel the distress or frustration of those they lead without being overwhelmed by the emotions and therefore unable to help (Gilbert, 2019);
- Helping: the helping element has four components: scope—the breadth of resources offered; scale—the volume of resources; speed—the timeliness of the response; and specialization—the extent to which the response meets the real needs of the other) (Lilius et al., 2011).

Two papers on this Research Topic show the relevance of leadership roles in promoting employees' happiness at work and in their personal lives. In the first paper, Wan et al. emphasize he crucial role of emotions at work, showing that leaders can influence subordinates' emotions through emotional contagion and emotional appeal, thus ultimately affecting their job performance. In other words, the greater followers' susceptibility to positive emotion, the greater the effect of positive emotional leadership on their positive emotions (Wan et al.). In the second study, Yao et al. examine leadership's impact on the work-family interface. These results provide further evidence of how leadership styleaffects employees' personal lives. They show that benevolent leaders may affect marital family satisfaction through the full mediation of work-family facilitation, while the negative relationship between authoritarian leadership and marital family satisfaction is fully mediated by work-family conflict (WFC).

Compassion at work has been mainly studies in healthcare systems, where workers are continuously challenged by the high relational content of their caring associated emotional demands However other studies have demonstrated the effectiveness of compassion in other organizational contexts, particularly its role in influencing individual wellbeing and productivity and in relation to its protective role against work-related stress and burnout (e.g., Tabaj et al., 2015; Eldor and Shoshani, 2016; Buonomo et al., 2022). Buonomo et al. paper addresses the role of compassion at school, given the saliency of caring in teaching as a helping profession. The findings show that compassion satisfaction, as a job resource, is strongly related to teachers' work engagement at school, confirming that teachers' care toward their students is a crucial resource supporting their engagement.

The research included in this Research Topic supports other evidence showing that compassion makes a profound difference at work. In this Compassion is not a personality trait; this means that it can be promoted and sustained by organizations at individual and group levels. Those who receive compassion from their colleagues or their manager tend to replicate it; thus, compassion can be learned and developed as an individual orientation and organizational culture.

All organizations (whichever types they are: non-profit and for-profit; governmental and private ones) can deliberately choose to change their culture, processes, and actions to develop compassion as a core value (West, 2021). This special issue aims to help and promote this transition, as an important component in efforts to improve work contexts to enable human growth and flourishing and organizational performance.

The paper by Andersson et al. shows the effects of a 6-week psychological intervention utilizing compassion training on stress, mental health, and self-compassion. The findings show promising results regarding the success of compassion training within organizations in decreasing stress and mental ill-health and increasing self-compassion.

In this Research Topic, we aimed to explore how compassion and compassionate leadership at work can help us ensure a more meaningful, pleasurable, and productive experience for employees, creating safe and positive work environments. We hope the papers presented in this special issue will help develop and disseminate commitment to this important area of research and practice.

## Author contributions

PB and IB wrote the draft of the Editorial. MW reviewed and edited the draft to finalize it. All the authors approved the submitted version of this paper.

## References

[B1] Atkins P. W. B. Parker S. K. (2012). Understanding individual compassion in organizations: The role of appraisals and psychological flexibility. Acad. Manag. Rev. 37, 4. 10.5465/amr.2010.0490

[B2] Buonomo I. Santoro P. E. Benevene P. Borrelli I. Angelini G. Fiorilli C.. (2022). Buffering the effects of burnout on healthcare professionals' health-the mediating role of compassionate relationships at work in the COVID era. Int. J. Environ. Res. Public Health. 19, 8966. 10.3390/ijerph19158966 35897337 PMC9332033

[B3] Eldor L. Shoshani A. (2016). Caring relationships in school staff: Exploring the link between compassion and teacher work engagement. Teach. Teach. Educ. 59, 126–136doi: 10.1016/j.tate.2016.06.001

[B4] Gilbert P. (2019). Explorations into the nature and function of compassion. Curr. Opin. Psychol. 28, 108–114. 10.1016/j.copsyc.2018.12.002 30639833

[B5] Lilius J. M. Worline M. C. Dutton J. E. Kanov J. M. Maitlis S. (2011). Understanding compassion capability. Hum. Relations. 64, 873–899. 10.1177/0018726710396250

[B6] Tabaj A. Pastirk S. Bitenc C. Masten R. (2015). Work-related stress, burnout, compassion, and work satisfaction of professional workers in vocational rehabilitation. Rehabil. Couns. Bull. 58, 113–123. 10.1177/003435521453738335487521

[B7] West M. Armit K. Loewenthal L. Eckert R. West T. Lee A.. (2015). Leadership and Leadership Development in Health Care: The Evidence Base London: The King's Fund.

[B8] West M. A. (2021). Compassionate Leadership: Sustaining Wisdom, Humanity and Presence in Health and Social Care. London: Swirling Leaf Press.

